# Complete genome sequences from clinical isolates of neonatal meningitis *Escherichia coli*

**DOI:** 10.1128/mra.00548-26

**Published:** 2026-07-10

**Authors:** Julia Ienes-Lima, Nicolle Lima Barbieri, Lisa K. Nolan, Catherine M. Logue

**Affiliations:** 1 Department of Population Health, College of Veterinary Medicine, University of Georgia 1355, Athens, Georgia, USA; 2 Department of Infectious Diseases, College of Veterinary Medicine, University of Georgia 1355, Athens, Georgia, USA; Fluxus Inc., Sunnyvale, California, USA

**Keywords:** genomes, *Escherichia coli*, neonatal meningitis

## Abstract

Neonatal meningitis *Escherichia coli* (NMEC) is the most common gram-negative microorganism to cause meningitis in newborns. Here, we report the complete genome sequences of 29 NMEC strains isolated from the cerebrospinal fluid of infants with meningitis in the Netherlands from 1989 to 1997.

## ANNOUNCEMENT

Neonatal meningitis *Escherichia coli* (NMEC) is the most common gram-negative microorganism to cause meningitis and sepsis in neonates worldwide (1, 2). Since the 1990s, NMEC has emerged as a causative agent of bacteremia and meningitis among very low birth weight and preterm infants (3–5). However, the availability of NMEC genomes remains scarce. Here, we present the complete genome sequences of 29 strains of NMEC, isolated from the cerebrospinal fluid of anonymous infants diagnosed with meningitis from 1989 to 1997, as previously described (6, 7).

The original isolates were stored in Luria Bertani (LB) broth with 25% glycerol at −80°C. Isolates were streaked with an inoculating loop from frozen stock onto LB agar and incubated overnight at 37°C. After incubation, genomic DNA was extracted using the MagAttract high-molecular-weight DNA kit (Qiagen, Hilden, Germany) and quantified using the Qubit Double-Stranded DNA High-Sensitivity Assay Kit (Life Technologies, Carlsbad, CA, USA). DNA quality was evaluated using a NanoDrop 2000 Spectrophotometer (Thermo Fisher Scientific, Waltham, MA, USA) and analyzed on an E-Gel SizeSelect 2% Agarose Gel (Invitrogen, Waltham, MA, USA). DNA was sheared to an average fragment size of 10–15 kb using the Covaris G-tube (Covaris Inc., Woburn, MA, USA) and confirmed using the Agilent 2100 Bioanalyzer (Agilent Technologies, Santa Clara, CA, USA). Libraries were prepared following the PacBio large-insert gDNA workflow using the SMRTbell Express Template Prep Kit v2.0 (Pacific Biosciences, Menlo Park, CA, USA). Library preparation included DNA damage repair, end repair, and barcoded adapter ligation, followed by nuclease treatment to remove non-ligated fragments. BluePippin Size Selection (Sage Science, Beverly, MA, USA) was used to target a library size of >7 kb.

Sequencing was performed on the PacBio Sequel II platform (Pacific Biosciences), generating circular consensus sequencing reads. Read quality was assessed using FastQC v0.11.9 (https://www.bioinformatics.babraham.ac.uk/projects/fastqc/) and SeqKit (8). Reads were assembled using Hifiasm v0.24.0-r707 (9), followed by a quality and completeness check using QUAST v5.2.0 (10) and BUSCO v5.8.3 (11), respectively. Minimap2 v2.28 (12) and SAMtools v1.21 (13) were used to assess genome depth and coverage. Assembled genome sequences were rotated to the default starting gene (*dnaA*) using Circlator v1.5.5 (14) and circularized in Geneious v2024.0.5 (15). Plasmids were identified using PlasmidFinder (16) database through ABRicate v1.0.1 (https://github.com/tseemann/abricate) and MOB-suite v3.1.9 (17). Default parameters were used for all software. Assembly statistics are described in Table 1.

**TABLE 1:** Assembly statistics of NMEC genomes deposited under BioProject PRJNA1383263

BioSample	SRA accession	GenBank accession	Strain	Total no. of reads	Reads N50 (bp)	No. of contigs	No. chrom. /plasmids contigs^*a*^	Assembly N50 (bp)	Total assembly length (bp)	GC content (%)	Genome coverage
SAMN54121275	SRR36640895	JBUBLZ000000000	N1	19,236	8,177	2	1/1	5,098,136	5,257,543	50.5	30×
SAMN54121276	SRR36640894	JBUBMA000000000	N10	41,071	8,554	6	1/5	5,123,235	5,399,687	50.5	65×
SAMN54121277	SRR36640882	JBUBMB000000000	N12	26,942	8,760	1	1/0	5,083,538	5,083,538	50.5	46×
SAMN54121278	SRR36640873	JBUBMC000000000	N17	24,399	8,752	1	1/0	4,857,330	4,857,330	50.5	44×
SAMN54121279	SRR36640872	JBUBMD000000000	N21	36,053	8,662	3	1/2	5,208,995	5,405,789	50.5	58×
SAMN54121280	SRR36640875	JBUBME000000000	N24	43,835	9,237	3	1/2	4,856,607	5,102,111	50.5	80×
SAMN54121281	SRR36640871	JBUBMF000000000	N25	25,576	8,387	7	1/6	5,000,240	5,264,565	51	40×
SAMN54121282	SRR36640870	JBUBMG000000000	N31	33,575	8,928	5	1/4	5,002,615	5,395,753	50.5	56×
SAMN54121283	SRR36640869	JBUBMH000000000	N32	22,583	8,810	3	1/2	5,233,447	5,354,977	50.5	38×
SAMN54121284	SRR36640868	JBUBMI000000000	N33	17,154	8,809	2	1/1	4,470,553	4,588,839	50.5	30×
SAMN54121285	SRR36640893	JBUBMJ000000000	N39	19,150	8,231	1	1/0	5,039,009	5,039,009	50.5	31×
SAMN54121286	SRR36640892	JBUBMK000000000	N41	16,470	8,067	3	1/2	5,208,887	5,345,304	51	24×
SAMN54121287	SRR36640890	JBUBML000000000	N44	22,926	8,474	3	1/2	5,129,373	5,381,508	50.5	35×
SAMN54121288	SRR36640889	JBUBMM000000000	N47	29,365	8,664	2	1/1	4,902,534	4,920,970	50.5	50×
SAMN54121289	SRR36640888	JBUBMN000000000	N48	17,320	8,156	2	1/1	5,080,239	5,276,741	50.5	27×
SAMN54121290	SRR36640891	JBUBMO000000000	N50	28,135	8,948	3	1/2	5,253,559	5,498,979	50.5	46×
SAMN54121291	SRR36640887	JBUBMP000000000	N51	20,559	8,309	3	1/2	5,222,106	5,382,993	50.5	31×
SAMN54121292	SRR36640886	JBUBMQ000000000	N53	22,866	8,102	3	1/2	5,070,021	5,229,260	50.5	35×
SAMN54121293	SRR36640885	JBUBMR000000000	N54	22,736	8,202	1	1/0	5,144,377	5,144,377	50.5	35×
SAMN54121294	SRR36640884	JBUBMS000000000	N56	23,131	8,160	3	1/2	5,061,883	5,315,958	50.5	35×
SAMN54121295	SRR36640881	JBUBMT000000000	N58	44,405	8,822	2	1/1	5,009,951	5,170,678	50.5	75×
SAMN54121296	SRR36640880	JBUBMU000000000	N61	15,356	8,135	2	1/1	5,096,546	5,254,499	50.5	24×
SAMN54121297	SRR36640883	JBUBMV000000000	N62	22,725	8,520	4	1/3	4,994,894	5,257,611	50.5	37×
SAMN54121298	SRR36640879	JBUBMW000000000	N63	17,930	7,982	4	1/3	4,991,345	5,271,478	50.5	27×
SAMN54121299	SRR36640878	JBUBMX000000000	N64	27,579	8,193	2	1/1	4,869,580	4,950,717	50.5	45×
SAMN54121300	SRR36640877	JBUBMY000000000	N66	14,213	7,816	4	1/3	4,998,827	5,311,965	50.5	21×
SAMN54121301	SRR36640876	JBUBMZ000000000	N68	20,374	8,197	2	1/1	5,177,290	5,278,055	50.5	31×
SAMN54416500	SRR36664837	JBTLPV010000000	N69	27,138	8,446	4	2/2	2,429,229	5,164,028	50.5	44×
SAMN54121302	SRR36640874	JBUBNA000000000	N9	27,888	9,093	1	1/0	4,727,862	4,727,862	51	54×

^*a*^ chrom., chromosome.

The 29 NMEC genomes presented an average length and GC content of 5,194,211 bp and 50.5%, respectively. Only one genome was not closed into a single chromosome (strain N69). Most of the strains harbor two or fewer plasmids (79%), while six (21%) strains harbor ≥3 plasmids. Chromosomes and plasmids were annotated using the National Center for Biotechnology Information (NCBI) Prokaryotic Genome Annotation Pipeline v6.10 (18) with the best-placed reference protein set and GeneMarkS-2+. The average number of predicted protein-coding sequences among the genomes was 4,982, encoding an average of 121 RNAs (22 ribosomal RNAs, 92 transfer RNAs, seven non-coding RNAs). Average nucleotide identity analysis was performed during QUAST quality check, and on average, 99.2% of the DNA sequences were identical to the representative NMEC strain RS218 CP007149.1 (19, 20).

## Data Availability

The genome sequences were deposited in the National Center for Biotechnology Information (NCBI) under BioProject accession number PRJNA1383263. Sequence Read Archive (SRA) and BioSample accession numbers are listed in Table 1.
